# Central nervous system disorders after use of dolutegravir: evidence from preclinical and clinical studies

**DOI:** 10.1007/s43440-023-00515-y

**Published:** 2023-08-21

**Authors:** Alicja Jakimiuk, Agnieszka Piechal, Alicja Wiercińska-Drapało, Alicja Nowaczyk, Dagmara Mirowska-Guzel

**Affiliations:** 1 Department of Clinical and Experimental Pharmacology, Centre for Preclinical Research and Technology, Medical University of Warsaw, Banacha 1b, 02-097 Warsaw, Poland; 2 Department of Hepatology and Infectious and Tropical Diseases, Medical University of Warsaw, Provincial Infectious Diseases Hospital in Warsaw, Wolska 37, 01-201 Warsaw, Poland; 3 Department of Organic Chemistry, Faculty of Pharmacy, Ludwik Rydygier Collegium Medicum in Bydgoszcz, Nicolaus Copernicus University in Toruń, 2 dr. A. Jurasza, 85-094 Bydgoszcz, Poland

**Keywords:** Dolutegravir, Central nervous system, Adverse effects, HIV, Antiretroviral drugs

## Abstract

The evaluation of dolutegravir based on available preclinical and clinical studies reveals a risk of central nervous system (CNS) disorders associated with long-term use of the drug. The available literature on the pharmacokinetics of the drug, including its penetration of the blood–brain barrier, was reviewed, as well as clinical trials assessing the incidence of adverse effects in the CNS and the frequency of its discontinuation. This paper also summarizes the impact of factors affecting the occurrence of CNS disorders and indicates the key role of pharmacovigilance in the process of supplementing knowledge on the safety of drugs, especially those that are newly registered.

## Introduction

As a result of damage to the blood–brain barrier (BBB) in human immunodeficiency virus (HIV)-infected patients, the virus invades the central nervous system (CNS) in the first few days, causing the development of a long-term inflammatory process and damage to the nerve cells. This leads to a variety of CNS disorders. Before the era of combined antiretroviral therapy (cART), HIV-associated dementia (HAD) and distal sensory polyneuropathy were experienced by as many as 35% of patients [1]. HAD now occurs in 2–5% of patients, and polyneuropathy has been almost completely eliminated [1]. Other neurological disorders include meningoencephalitis, Guillain-Barré syndrome, polymyositis, transverse myelitis, cranial or peripheral nerve damage, and psychiatric disorders, including anxiety and restlessness, depressive and psychotic disorders, and sleep disorders [2].

Drugs that penetrate well into the CNS are used to reduce the risk of central symptoms in HIV patients, leading to a reduction in HIV RNA in the cerebrospinal fluid (CSF). However, the use of increasingly effective antiretroviral drugs (ATRs) may also affect the CNS and cause adverse effects; the occurrence of neuropsychiatric disorders may lead to discontinuation of treatment [3].

The problem of neurotoxicity may be linked to many groups of ATRs. Such reports have appeared in the case of dolutegravir (DTG), one of the most commonly used drugs belonging to a new class of ATRs prescribed to patients infected with HIV-1. DTG is a second-generation integrase chain transfer inhibitor (INSTI), interrupting an enzyme involved in the reproduction of HIV. DTG works by blocking the transport and incorporation of proviral DNA into the host T-cell genome, inhibiting further steps in the replication process. This slows the multiplication and spread of the virus [4]. DTG was registered for the first time on August 12, 2013, by the Food and Drug Administration (FDA), and it was approved for marketing by the European Medicines Agency (EMA) on January 16, 2014, for both previously untreated and treated patients, including patients with resistance to integrase inhibitors [5].

The INSTI group also includes raltegravir (RAL), elvitegravir (EVG), bictegravir (BIC), and cabotegravir (CAB). Clinical trials in which oral INSTIs were used showed high efficiency with a rapid decrease in HIV RNA already in the fourth week from the start of treatment. In a study that compared the effectiveness of DTG with BIC, 76–80% of ATR-naïve HIV-positive patients presented with virological suppression as early as 4 weeks after starting [6–10]. The INSTIs, including DTG, are well tolerated and considered safe and recommended as first-line drugs in HIV-positive patients as part of combination therapy with other ATRs [10–12].

## Selected pharmacokinetic parameters of dolutegravir in preclinical studies

**Fig. 1:**
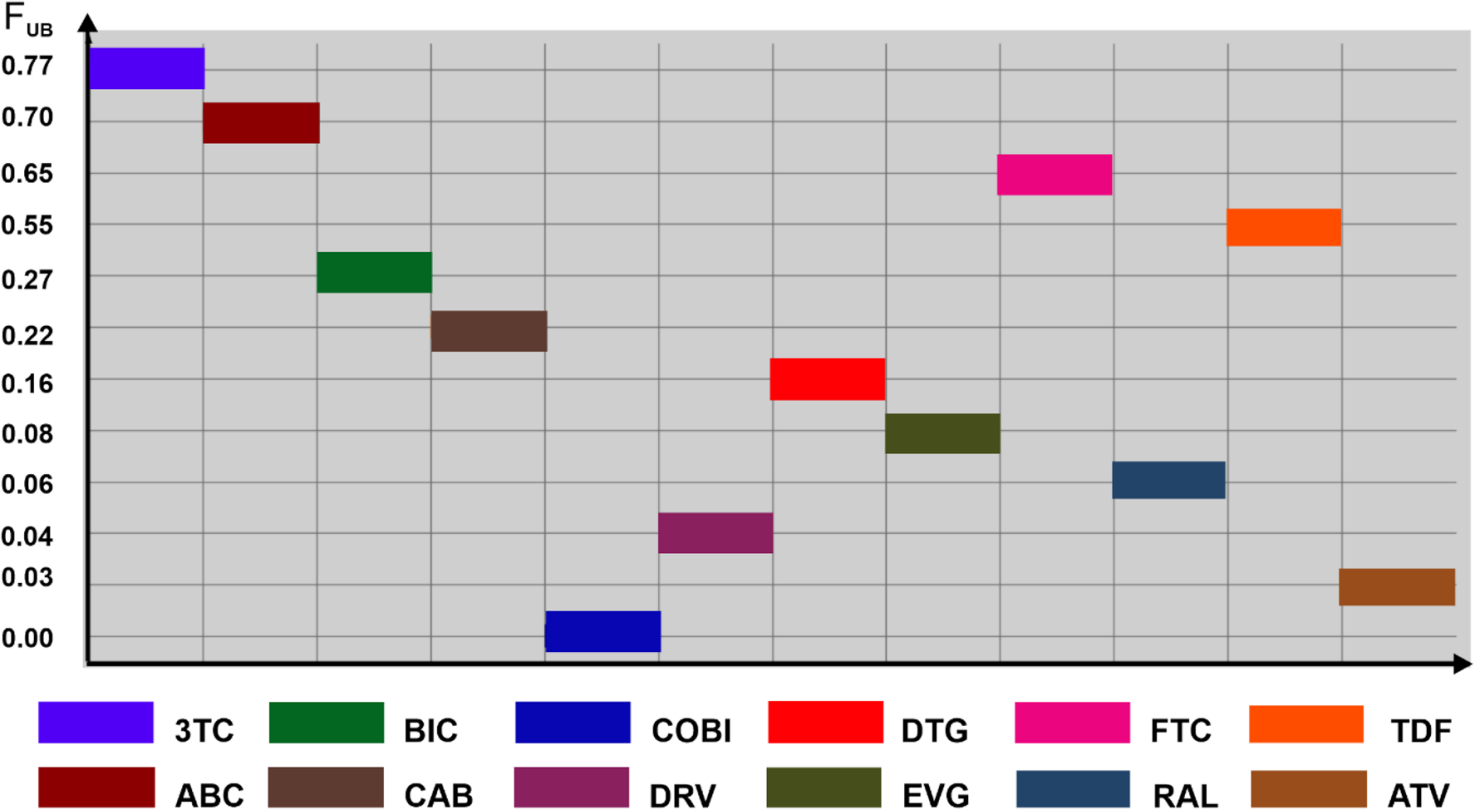
A predictive model exhibiting the fraction unbound to proteins in the blood (F_UB_). Drugs studied: *3TC* lamivudine, *ABC* abacavir, *BIC* bictegravir, *CAB* cabotegavir, *COBI* cobicistat, *DRV* darunavir, *DTG* dolutegravir, *EVG* elvitegravir, *FTC* emtricitabine, *RAL* raltegravir, *TDF* tenofovir, *ATV* atazanavir

A few preclinical studies have evaluated the neurotoxicity of DTG. Moss et al. [13] revealed the presence of DTG in the brain up to 10 h after administration of a single dose of 50 mg/kg [^14^C] DTG to male rats. However, DTG radioactivity was low (< 2% of blood radioactivity). A recent mouse pharmacokinetics study of tenofovir disoproxil fumarate (TDF), emtricitabine (FTC), and DTG showed that each of the drugs had low brain exposure, with only TDF achieving concentrations above the 90% inhibitory concentration (IC_90_) [14]. According to the authors, the low concentration of DTG in the brain may be the result of a low degree of non-binding (F_UB_) to plasma proteins, limiting diffusion through the BBB (Fig. 1).

The effectiveness of a given drug depends on the state of equilibrium between the unbound and bound state of the drug in plasma. The more of the free drug present in the serum, the more effectively it penetrates or diffuses the cell membranes [15]. Studies conducted in pregnant mice have shown that DTG easily reaches the CNS and inhibits the activity of matrix metalloproteinases [16]. In another study, Hinckley et al. [17] showed that DTG has a small but significant effect on neuronal growth.

## Bioavailability

Focusing on issues other than pharmacodynamic efficacy, numerous therapeutic failures correspond directly to poor bioavailability of the drug. Absorption in the gastrointestinal tract and passage through the BBB are two pharmacokinetic processes that should be taken into consideration during pharmacotherapy. Though there are various routes of drug administration, oral administration is highly preferred due to comfort and patient compliance. Early estimation of oral bioavailability (i.e., the fraction of the dose that reaches the bloodstream) following oral administration is a key criterion for making therapeutic decisions. Bioavailability depends on many factors, including absorption in the gastrointestinal tract.

**Fig. 2:**
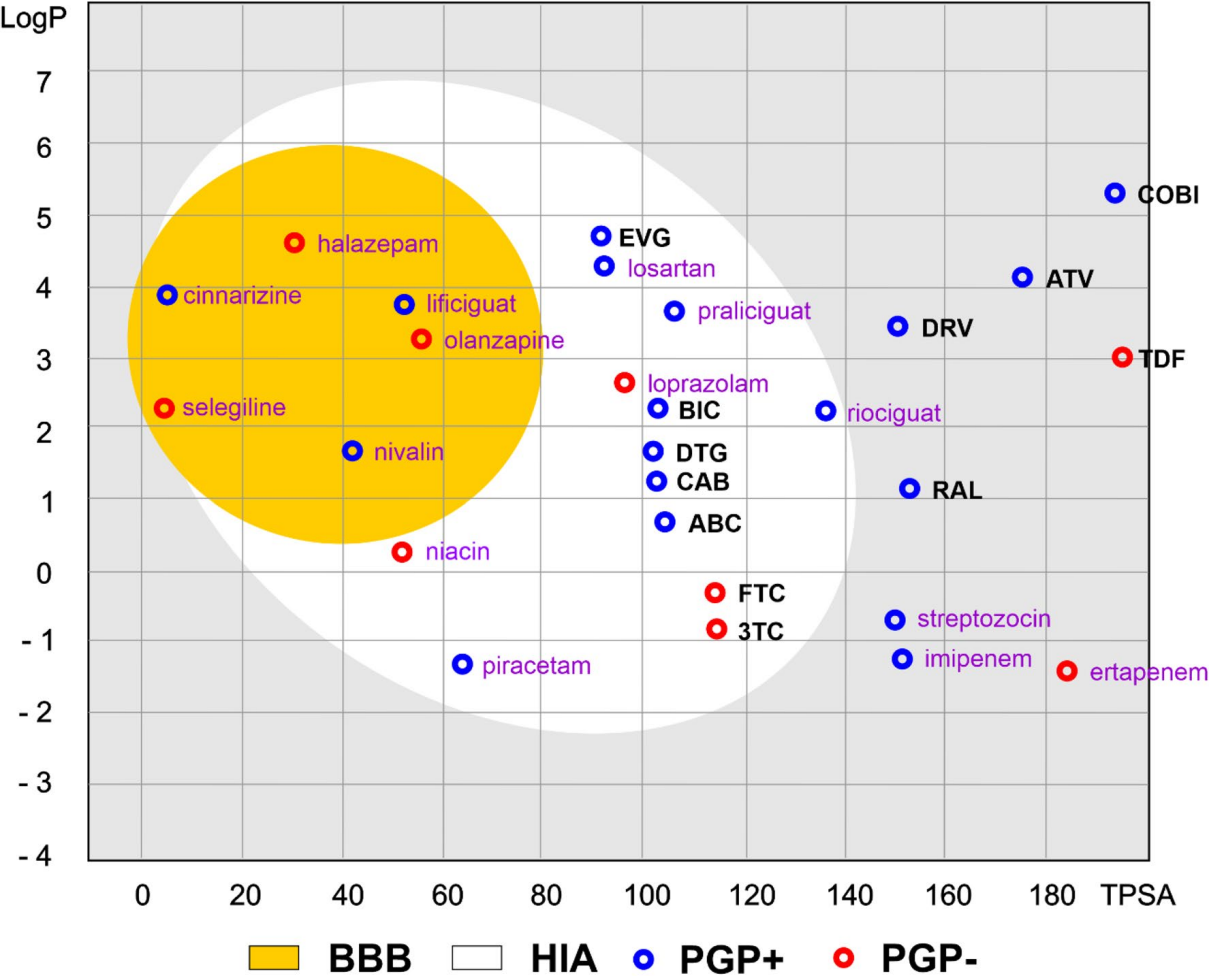
The Egan egg chart compares the study drugs and selected commonly used drugs. Drugs studied: *3TC* lamivudine, *ABC* abacavir, *BIC* bictegravir, *CAB* cabotegavir, *COBI* cobicistat, *DRV* darunavir, *DTG* dolutegravir, *EVG* elvitegravir, *FTC* emtricitabine, *RAL* raltegravir, *TDF* tenofovir, *ATV* atazanavir. The colored areas represent the optimal prediction range (above 93%) of penetration for the brain (BBB, yellow) and gastrointestinal tract (HIA, white). Gray indicates the area where penetration into the brain and gastrointestinal tract may occur, but its probability is below the optimal value. A molecule predicted to be effluated from the CNS by P-glycoprotein (PGP + , blue dots) or not (PGP-, red dots). The names of the compounds are indicated in different font colors, i.e., black corresponds to the drugs tested, and purple corresponds to the selected drugs for comparison. Prognostic data were obtained using pkCSM pharmacokinetics [15, 18]

Egan et al. [18] developed a routinely used prediction tool to discriminate between well and poorly absorbed molecules based on the 2D plane of drug physicochemical parameters. By analyzing lipophilicity [described by the n-octanol/water partition coefficient (log P)] and polarity [determined by the polar surface area index (PSA)], the proposed model estimates the probability of molecule absorption. Due to the most likely area of absorption being elliptical in shape, it has been called the BOILED-Egg (Brain Or IntestinaL Estimate D permeation method) [19, 20]. Figure 2 shows the Egan egg graph comparing the tested ATRs and selected commonly used drugs. Colored areas represent the optimal prediction range (above 93%) for the brain (BBB, yellow) and gastrointestinal [human intestinal absorption (HIA), white] penetration. Gray represents the area where brain and gastrointestinal penetration can occur, but the probability is significantly below the optimal value.

DTG (similar to EVG, BIC, CAB, ABC, FTC, 3TC; Fig. 2) is characterized by a high probability of absorption from the gastrointestinal tract. However, none of the analyzed drugs penetrate the BBB, confirming their low penetration of the CNS. Labarthe et al. showed that DTG is a substrate for the efflux transporters ABCB1 and ABCG2 [mainly P-glycoprotein (PGP)] present in the BBB [14]. However, Tisseraud et al. [21] showed a very low positron emission tomography (PET) signal in the brains of macaques in a PET imaging study using [^18^F]DTG, which also suggested a low penetration of DTG into the CNS. PGP acts as a biological barrier; therefore, its substrates (toxins and xenobiotics) are excreted from cells, and inhibitors imply specific adverse effects. The presented analysis (Fig. 2) indicates whether a given compound can be a substrate for PGP. Notably, DTG is cleared from the CNS by PGP, which provides another explanation as to why DTG has poor brain penetration in animal studies [21].

## DTG penetration of the brain barrier

Biological barriers provide protection against invasion by pathogens and diseases, but also complicate drug delivery [22]. The adult human brain has five barrier interfaces that regulate molecular traffic into the brain parenchyma: the BBB [23, 24], blood–cerebrospinal fluid barrier [25], blood–arachnoid barrier [26], the circumventricular organs [27], and ependyma [28]. The barriers between the blood and extracellular matrix of the brain form tight endothelial cell (EC) structures joined together by protein junctions. The BBB is formed by the ECs lining the cerebral microvessels and separates the blood from the interstitial fluid of the brain [29]. The choroid plexus epithelium sits between the blood and the ventricular CSF and forms the blood–CSF barrier. The arachnoid barriers are formed by the epithelium sandwiched between the blood and the subarachnoid CSF. These three barrier layers participate in limiting and regulating molecular exchange at the interface between the blood and nervous tissue or its fluid spaces [30]. The biological barriers’ intrinsic functions affect both drug delivery and uptake, hindering effective therapeutic outcomes. In addition to hindering treatment options, they also reduce the bioavailability of drugs in areas protected by the barriers, which can ultimately lead to increased drug resistance. On the other hand, inappropriate intervention at these barriers can disrupt their natural functions, increasing the risk of infection or opening channels for pathogens [19]. The PGP protein co-creates cell barriers and is a protein membrane transporter that actively removes harmful substances from cells. PGP localizes inside important organs, such as the brain, placenta, liver, intestine, and kidneys, where it plays a role in the distribution and elimination of drugs from the body. PGP is also found in capillary ECs that function as blood–brain, blood–testis, and blood–placental barriers [31]. The protein forms a transmembrane one-way efflux pump utilizing ATP in active transport of substances from cells against their concentration gradients. PGP has also been shown to be strongly involved in multidrug resistant diseases [32]. This seems to be important in the treatment of HIV infection [34]. In most tissues, PGP is present on the cell’s free surface, facing the lumen of the vessels. This location indicates its most important function, preventing the penetration of xenobiotics (mainly drugs) into the nervous tissue in the brain via removal of xenobiotics from the ECs back into the blood. Thus, PGP influences the pharmacological profile of numerous substances and their metabolites, as it alters their oral bioavailability, absorption in certain tissues, and elimination from the body [33]. As a multidrug transporter, PGP is characterized by broad substrate specificity, as it recognizes a very large number of compounds of various chemical structures and molecular weights (from 330 to 4000 Da). PGP transports hydrophobic or neutral substances or cations, but not anions. The log P ≈ 2.2 for DTG [34] means that it is a medium hydrophobic substance that is only partially bioaccumulating [35]. Numerous pharmacological studies have shown that DTG is immediate pumped back into the blood by PGP when it enters the ECs as a substrate [14]. Disruption of the BBB barrier by HIV causes PGP dysfunction, which contributes to easier penetration of drugs, including DTG, into brain tissues [36]. The resulting increase in the concentration of DTG in the brain results in the intensification of undesirable effects, such as insomnia and headache [37]. Taking into account the presence of PGP in tissues performing efflux function (small intestine, liver, and kidneys), dysfunction of the protein will increase pathological symptoms. Recent research suggests that PGP initiates the production of T effector cells after viral infection, whereas PGP has a protective function against T memory cells in the case of bacterial invasion [38].

## DTG penetration of the blood–brain barrier in clinical trials

Studies conducted in a group of 13 HIV-infected patients showed that the concentration of DTG was lower in the CSF (median 9.6 ng/mL; range 3.6–22.8 ng/mL) than serum (median 1675 ng/mL; range 3137–5091 ng/mL) and may be comparable to the concentration of non-protein-bound DTG (median 9.2 ng/mL; range: 0.8–34.5 ng/mL) [39]. In all patients, the concentration of DTG in CSF was above the IC_50_ (0.2 ng/mL) assessed in vitro [40] and above the therapeutic concentration (~ 2.4 ng/mL) [41]. However, the concentration of DTG in the CSF did not correlate with the total concentration of DTG in serum and the concentration of DTG unbound to proteins. The transfer of DTG to the CSF positively correlated (*r* = 0.6396, *p* = 0.0186) with the quotient of serum albumin concentration to CSF concentration. The authors suggest that DTG enters the CSF by diffusion, and DTG diffusion into the CSF increases with increased permeability of the BBB [39].

The concentration of DTG in CSF was also assessed by Letendre et al. [42]. The authors showed that the median concentration of DTG in CSF was 18 ng/mL (range 4–23 ng/mL) in week 2 of treatment and 13 ng/mL (4–18 ng/mL) in week 16. The concentration of DTG in CSF was comparable to unbound DTG in plasma. In week 2 of treatment, the median CSF concentration of DTG was more than 90-times higher, and in week 16 more than 66-times the IC_50_. At the same time, after 16 weeks, the number of HIV RNA copies was < 50 copies/mL, which indicates high antiretroviral activity. Calgagno et al. showed that people > 50 years of age had higher concentrations of DTG in CSF and a higher CSF-to-serum ratios [43].

Yagura et al. studied the concentration of DTG in the CSF of 162 Japanese patients. In 41 of the examined patients, CNS disorders (e.g., dizziness, headache, restlessness, and anxiety) occurred and the concentration of DTG in CSF was higher than the concentration of the drug in patients without neuropsychiatric adverse events (NPSAEs) [44].

The serum and CSF concentrations of DTG and selected ATRs are presented in Table 1.

**Table 1:** Serum and CSF concentrations of selected antiretrovirals

Drug	Serum concentration	Concentration in CSF	Other	References
Integrase strand transfer inhibitor (INSTI)				
DTG	1675 ng/mL	9.6 ng/mL	The drug enters the CSF by diffusion and its concentration increases as the blood–brain barrier increases	[39]
	2 weeks: 3360 ng/mL 16 weeks: 3210 ng/mL	2 weeks: 18.2 ng/mL 16 weeks: 13.2 ng/mL	Total CSF-to-plasma ratio 0.41%	[42]
RAL	448 ng/mL	18.4 ng/mL		[45]
	165 ng/mL	31 ng/mL	Concentration of RAL depends on the permeability of the blood–brain barrier	[46]
	260.9 ng/mL	14.5 ng/mL	CSF concentration correlates with serum concentration	[47]
EVG	676–1.389 ng/mL	2.4–11.7 ng/mL	Tested on three patients	[48]
BIC	1131.5–4781.1 ng/mL	7.12–20.16 ng/mL	Tested on six patients	[49]
	1837.1 ng/mL	6.9 ng/mL 2.48 ng/mL (unbound fraction)		[50]
	2610 ng/mL	11.8 ng/mL 4.4 ng/mL (unbound fraction)		[51]
CAB	Q8W 3920 ng/mL 4.7 ng/mL (unbound drug) Q4W 3020 ng/mL 4.7 ng/mL (unbound drug)	Q8W 10.6 ng/mL Q4W 12.7 ng/mL	Total CSF-to-plasma ratio 0.30% to 0.34%	[52]
Nucleoside reverse transcriptase inhibitor (NRTI)				
ABC	139 ng/mL	128 ng/mL	T_1/2_ in CSF > 2-times higher than in serum (2.5 ± 0.6 h vs. 1.2 ± 0.2 h)	[53]
			GpP limits ABC penetration to OUN	[54]
	1 time daily: 96 ng/mL 2 times daily: 22 ng/mL	123 ng/mL 49 ng/mL		[55]
TDF	49 ng/mL	6 ng/mL	CSF-to-plasma ratio 0.05 (0–0.13)	[56]
		Not detected		[48]
	51.5 and 53.1 ng/mL	Not detected		[57]
	19.7 ng/mL	1.6 ng/mL ND (unbound fraction)		[51]
FTC	212 ng/mL	68 ng/mL	CSF-to-plasma ratio 0.26 (0.05–0.41)	[56]
	158 ng/mL	84.4 ng/mL ND (unbound fraction)		[51]
3TC	67.75 ng/mL	43.42 ng/mL	CSF-to-plasma ratio 0.417	[58]
Non-nucleoside reverse transcriptase inhibitor (NNRTI)				
EFV	3718 ng/mL	16.3 ng/mL	CSF-to-plasma ratio 0.0044	[59]
	2170 ng/mL	18.8 ng/mL		[60]
	2145 ng/mL	13.9 ng/mL	CSF-to-plasma ratio 0.005	[61]
RPV	Q8W 192 ng/mL Q4W 134 ng/mL	Q8W 1.84 ng/mL Q4W 1.67 ng/mL	CSF-to-plasma ratio 1.07% to 1.32%	[52]
		1.54 ng/mL	CSF-to-plasma ratio 0.97%	[62]
Protease inhibitor				
ATV	523 ng/mL	7.9 ng/mL	Less than 1% penetration into CSF	[63]
	1250 ng/mL	8.3 ng/mL	CSF/plasma 0.9%	[64]
	295.8 ng/mL (geometric mean)	8.7 ng/mL (geometric mean)	CSF/plasma 0.9%	[65]
DRV	3930 ng/mL	34.2 ng/mL	CSF/plasma 0.9%	[66]
	4094 ng/mL (total) 538 ng/mL (unbound)	55.8 ng/mL (total) 50.2 ng/mL (unbound)	CSF/plasma 0.014	[67]
	1907 ng/mL (geometric mean)	8.5 ng/mL (geometric mean)	CSF/plasma 0.005	[65]
		6.55 ng/mL	CSF/plasma 0.785	[62]

Q8W: cabotegravir LA 600 mg + rilpivirine LA 900 mg IM every 8 weeks

Q4W: cabotegravir LA 400 mg + rilpivirine LA 600 mg IM every 4 weeks

Drugs studied: *3TC* lamivudine, *ABC* abacavir, *BIC* bictegravir, *CAB* cabotegravir, *DRV* darunavir, *DTG* dolutegravir, *EVG* elvitegravir, *EFV* efavirenz, *FTC* emtricitabine, *RAL* raltegravir, *TDF* tenofovir, *ATV* atazanavir, *RPV* rilpivirine

## Neuropsychiatric disorders in clinical observations

The most common adverse effects observed in patients who started DTG therapy were nausea (13%), diarrhea (18%), and headache (13%). Other common (≥ 1/100 to < 1/10) effects include insomnia, abnormal dreams, depression, anxiety, dizziness, vomiting, flatulence, abdominal pain/upper abdominal pain, abdominal discomfort, rash, pruritus, feeling fatigue, and increased enzymes [alanine aminotransferase (ALT) and/or aspartate aminotransferase (AST), creatine phosphokinase (CPK)] [68].

It should be emphasized that DTG is characterized by high antiviral efficacy. One of the factors limiting its use is the occurrence of neuropsychiatric adverse events (NPSAEs), which is associated with a reduction in the effectiveness of treatment and, in extreme cases, discontinuation of therapy [69].

In 2017, Hoffman et al. [70] estimated the frequency of NPSAEs leading to discontinuation of therapy among patients treated with INSTIs in two German outpatient clinics in 2007–2016. Discontinuation rates due to adverse events occurring within 2 years of starting treatment with DTG, RAL, or EVG (with cobicistat—COBI, TDF—tenofovir, and FTC—emtricitabine) were compared. Factors affecting the discontinuation of DTG were also analyzed. The following neuropsychiatric disorders were assessed in the study: insomnia, sleep disturbances, dizziness, nervousness, anxiety, depression, decreased concentration, slow thinking, and unexplained pain or paresthesia. The rates of NPSAEs leading to discontinuation at 12 and 24 months were 5.6% and 6.7% for DTG, 0.7% and 1.5% for EVG, and 1.9% and 2.3% for RAL, respectively (i.e., more often related to DTG than other drugs in this class). NPSAEs leading to DTG discontinuation were observed more frequently in female patients, in patients over 60 years of age, and in HLA-B*5701-negative patients who started abacavir treatment at the same time. The NPSAEs (DTG vs. EVG/COBI/TDF/FTC vs. RAL) included insomnia and sleep disorders (36 vs. 2 vs. 4), attention deficit disorder (8 vs. 0 vs. 0), dizziness (13 vs. 1 vs. 3) headaches and paresthesia (16 vs. 1 vs. 6), and depression (7 vs. 0 vs. 1) [70].

Since then, many reports have been published on the safety of DTG. They showed that DTG has a favorable profile, but neurological and mental disorders may occur in patients a few months after the start of therapy, leading to discontinuation of DTG treatment. In the vast majority of cases, the severity of an NPSAE did not pose a threat to the patient’s life and did not require hospitalization. The NPSAEs resolved rapidly after discontinuation of DTG [71]. The discontinuation rate of DTG therapy due to neurotoxicity ranged on average from 2 to 10% [71, 72]. An example of the frequency of NPSAEs associated with the use of various ATRs is presented in Table 2.

**Table 2:** Incidence of NPSAEs in patients treated with DTG and other antiretroviral drugs (ARTs)

References	NPSAE	
	DTG (single drug or combination therapy)	Other ART
[70]	DTG: 7.45%	EFG: 1.16% RAL: 1.8%
[69]	DTG + 2NRTIs^‡^: 18.24%	RAL + 2NRTIs^‡^: 17.3%
[69]	DTG + 2NRTIs^‡^: 21.9%	DRV/r + 2NRTIs^‡^: 15.7%
[69]	DTG + ABC/3TC: 33.09%	EFV/TDF/FTC: 31.74%
[69]	ABC/DTG/3TC: 10.88%	ATV/r + TDF/FTC: 12.55%
[69]	DTG + ISBR:9.52%	RAL + ISBR: 8.56%
[73]	DTG + FTC/TAF: 23.4%	BIC/FTC/TAF: 24.6%
[74]	DTG: 1.69%	RAL: 0.62%
[75]	DTG: 8.2%	EFV: 25%
[76]	DTG/TDF/3TC: 16.9	EFV/TDF/3TC: 37.5%

*DTG* dolutegravir, *RAL* raltegravir, *ABC* abacavir, *3TC* lamivudine, *EFV* efavirenz, *TDF* tenofovir, *ATV* atazanavir, *FTC* emtricitabine, *TAF* tenofovir alafenamide, *BIC* bictegravir, *EVG* elvitegravir, *ISBR* investigator-selected background regimen

^‡^ABC/3TC or TDF/FTC

In 2017, Fettiplace et al. conducted a large-scale study assessing the incidence of NPSAEs in HIV-infected patients during treatment with DTG or other classes of antiretrovirals, including protease inhibitors (PIs) atazanavir (ATV) and darunavir (DRV), non-nucleoside reverse transcriptase inhibitor (NNRTI) efavirenz (EFV), and INSTI raltegravir. The analysis was based on data from five randomized phase III clinical trials (SPRING-2, FLAMINGO, SINGLE, ARIA, and SAILING) in which patients received DTG at a dose of at least 50 mg daily, the Observational Pharmaco-Epidemiology Research & Analysis (OPERA) cohort, and among cases spontaneously reported to the drug manufacturer. The assessed psychiatric disorders included different types of insomnia (insomnia, initial insomnia, terminal insomnia, and intermediate insomnia), anxiety (anxiety, anxiety disorder), depression (reported as depression, major depression, depressed mood, depressive symptoms, and bipolar disorder), and suicidal behavior (defined as suicide attempt, suicidal ideation, completed suicide, intentional self-harm, and self-injurious behavior). A low incidence of NPSAEs was observed in all five clinical trials but were most frequently reported by SINGLE (DTG: 17%), followed by SPRING-2 (DTG: 6%), SAILING (DTG: 3%), FLAMINGO (DTG: 8%), and ARIA (DTG: 4%). The severity of most symptoms was rated as mild or moderate [69].

A low rate of NPSAEs was also observed in the OPERA cohort study. The assessment included NPSAE diagnoses that occurred after treatment initiation, regardless of whether the patient had a prior diagnosis of a psychiatric disorder, and the rate of new NPSAEs that occurred in patients with no history of neuropsychiatric disorder at baseline or earlier. The follow-up period was similar to the other studies approximately 15 months. In the group of patients with a history of neuropsychiatric disorders, symptoms of anxiety, depression, or insomnia were most common in patients treated with DTG and least common in patients treated with EFV. In patients with no history of neuropsychiatric disorders, the rates of anxiety, depression, and insomnia were similar for all four comparators. In both the clinical trials and the OPERA cohort, NPSAEs were rare in patients treated with DTG. Furthermore, the rate of spontaneous reporting was low (clinical trials *N* = 3353; DTG, *n* = 1672; comparator therapies, *n* = 1681) in terms of estimated patient-years exposure time and not significantly different from clinical trial data [69].

Gallant et al. presented the results of a phase III randomized clinical trial comparing the efficacy and safety of a 48-week combination therapy containing DTG/abacavir (ABC)/lamivudine (3TC) or BIC/FTC/tenofovir (TAF) used in one tablet. Adverse reactions were reported more frequently in patients taking DTG/ABC/3TC, with the most common being nausea (BIC/FTC/TAF vs. DTG/ABC/3TC: 10.2% vs. 22.9%), headache (11.5% vs. 13.7%), and sleep disorders (4.5% vs. 6.3%) [77]. Another study involved HIV-infected patients not yet taking ATRs. During the 48-week therapy, patients were assigned to two receive one of two treatments: BIC/FTC/TAF or receiving FTC/TAF. The results of the above study were slightly different and did not indicate a higher incidence of neuropsychiatric disorders associated with DTG use. The most common adverse reactions (BIC/FTC/TAF vs. DTG/FTC/TAF) were headache (12.5% vs. 12.3%), diarrhea (11.8% vs. 12.0%), and nausea (7.8% vs. 9.5%). Adverse reactions leading to discontinuation of the study drug were rare and occurred in only 1 of 325 patients (< 1%) receiving DTG/FTC/TAF and as many as 5 of 320 patients (~ 1.5%) receiving BIC/FTC/TAF [73].

Llibre et al. presented the results of two phase III clinical trials, SWORD 1 and 2, which evaluated switching from triple or quadruple antiretroviral therapy to DTG (50 mg) plus RPV (25 mg) once daily in adults infected with HIV-1. Changing the once-daily regimen of DTG + RPV was highly effective and the outcome not worse than continuing triple or quadruple therapy. Although slightly more adverse events leading to treatment discontinuation occurred with DTG + RPV than with continued three- or four-drug therapy, they still occurred in a small percentage of patients; 2% of those taking DTG + RPV had discontinuation due to NPSAEs, such as anxiety, depression, depressed mood, insomnia, suicidal thoughts, and headaches. No increased risk of virologic failure was observed when switching to DTG from RPV once daily [78].

Cuzin et al. performed another analysis of data from 18 centers in France participating in the Dat’AIDS cohort study in which HIV-positive patients initiated INSTI treatment. All reasons for discontinuation of an INSTI-containing regimen were tracked, and the characteristics of patients discontinuing due to NPSAEs were described. Among the drugs used were DTG, as well as EVG administered together with COBI or RAL. The rate of NPSAEs leading to discontinuation was 2.7% for DTG, 1.3% for EVG, and 1.7% for RAL. Based on the analysis, DTG led to fewer virological failures (< 1%) than other INSTIs, was well tolerated, and showed high virological efficacy. On the other hand, discontinuation due to NPSAEs was reported in 2.7% of patients receiving DTG. Patients treated with DTG were at higher risk of developing NPSAEs than those treated with EVG or RAL in combination with COBI [79].

Peñafiel et al. conducted a retrospective analysis of a prospectively followed cohort of all antiretroviral-naïve and all virologically suppressed antiretroviral patients prescribed a first regimen of RAL, EVG, or DTG and had at least one follow-up visit. Early discontinuation for any reason was 271 per 1000 patient-years for RAL, 168 per 1000 patient-years for EVG, and 264 per 1000 patient-years for DTG (*p* = 0.0821). Adverse reactions leading to treatment discontinuation were mainly neuropsychiatric, musculoskeletal, or gastrointestinal disorders, and the most commonly reported neuropsychiatric symptoms were insomnia, dizziness, headache, and anxiety, the incidence of which was not significantly different between INSTI-treated patients. Particular NPSAEs leading to early treatment discontinuation were insomnia, dizziness, and headache. Discontinuation due to NPSAEs was more common with DTG than with RAL or EVG (*p* = 0.0046) [80].

In 2014–2016, a retrospective analysis was carried out of records of HIV-infected patients in the Netherlands. They checked the cause and time of discontinuation of DTG, which was included for both previously untreated and already treated patients. The average duration of DTG therapy was 225 days. Discontinuation of DTG therapy was observed in 85 patients (15.3%), and in 76 patients (13.7%), the reason for discontinuation was drug intolerance. Insomnia and sleep disorders were reported in 5.6% of patients, and neuropsychiatric disorders (*e.g*., anxiety, psychosis, and depression) occurred in 4.3% of patients. In patients receiving DTG in combination with ABC, discontinuation of DTG was reported even more often, with 58 patients (16.3%) discontinuing treatment, and the reason for discontinuation was of adverse effects [72].

In a prospective cohort study, Elzi et al. showed that DTG (1.7%) caused more neurotoxic effects than RAL (0.6%) and was more often the cause of treatment discontinuation. The authors attributed the slightly lower rates of CNS disorders compared to other studies to the smaller number of patients included in the study [74].

The frequency and reasons for discontinuation of treatment were also assessed by Fernández-Bargiela et al. in patients receiving DTG and EFV. Patients receiving EFV (35.8%) discontinued therapy more often than patients receiving DTG (12.1%), and the most common cause was NPSAEs (DTG, 8.2%; EFV, 25%). Women and those with documented psychiatric events were more likely to discontinue treatment. Furthermore, patients treated with DTG were less likely to be prescribed benzodiazepines. Both groups of patients required consultation and observation in psychiatric wards (DTG, 8.9%; EFV, 16.9%) [75].

In a cohort study by Mendes et al., DTG was found to have a better safety profile than EFV. Overall, 16.9% of CNS-related adverse events occurred in the group of patients receiving DTG/TDF/3TC, and NPSAEs were more than twice as frequent (37.5% of patients) in the group receiving EFV/TDF/3TC. Unexpectedly, alcohol consumption was associated with a lower risk of adverse events. The authors explained this phenomenon by the possibility of alcohol competing with cytochrome enzymes, a change in the metabolism of ATRs, a decrease in their plasma concentration, and a subsequently lower risk of adverse effects. Acute intoxication of the body may also occur after drinking alcohol, which manifests nausea and headaches, among other symptoms. These symptoms are similar to the adverse effects of medications. People who consume alcohol may attribute their symptoms to the effects of alcohol and, consequently, the number of adverse reactions reported was lower than in real life [76].

A recent cohort study conducted in a group of previously untreated and treated patients showed that the majority of patients (84%) discontinued DTG within the first 12 months of treatment, and the most common reason for discontinuation of therapy (92.2%) was CNS disorders. The probability of maintaining DTG treatment was 75.1% after 3 years and 67.2% after 5 years. A higher risk of treatment discontinuation was found in previously untreated patients. Patients who had a longer duration of virological suppression and were at risk of prior virological failure had a lower risk of treatment discontinuation [81].

In 2022, Taramasso et al. published the results of the prospective, observational SCOLTA cohort, which assessed the incidence of CNS adverse events after the administration of DTG and DTG-free ARTs. A total of 4939 HIV-infected subjects were enrolled in the study, of which 1179 were in the DTG group and 3760 in the non-DTG group [lopinavir/ritonavir (LPV/r, *n* = 731), atazanavir/ritonavir (ATV/r, *n* = 616), DRV/ritonavir or DRV/cobicistat (DRV/r or DRV/c, *n* = 721), RPV (*n* = 481), RAL (*n* = 514), EVG (*n* = 339), and BIC (*n* = 358)]. However, 834 (16.9%) had not received prior ART, whereas the remaining 4,105 had previously used ART, 2289 (55.8%) of which had < 50 copies HIV RNA/mL at the time of initiating the study drug. There was no significant difference in the incidence of neuropsychiatric disorders between the two cohorts at baseline, with 66 NPSAEs reported to lead to ART discontinuation, 39 (of 1179; 3.3%) in the DTG cohort and 27 (of 3760; 0.7%) in the non-DTG. HIV-infected, ART-naïve individuals with higher CD4 + T-cell counts and psychiatric disorders were more likely to develop CNS adverse events; non-NPSAEs were reported in 35/39 patients on DTG and 23/24 on non-DTG therapy. However, most NPSAEs were reversible and resolved when the ART was switched to a drug of the same or different class. At the same time, a lower event resolution rate was found in HIV-infected patients older than 50 years of age (*p* = 0.017). Thus, NPSAEs leading to discontinuation of ART occurred more frequently in patients treated with DTG than in those not treated with DTG. Most NPSAEs resolved after switching drugs, for both the DTG and non-DTG cohorts [82].

The impact of initial mental conditions on the occurrence of neuropsychiatric disorders after DTG use is unclear due to divergent information among HIV/AIDS specialists. Chan et al. showed that approximately 37 weeks of DTG use may be associated with an increased risk of moderate, but not severe, depressive symptoms [83]. However, the diagnosis of depression before the start of DTG therapy was not associated with the severity of disease symptoms. Povar-Echeverría et al. reported that patients treated with DTG who had a history of psychiatric disorders more often reported NPSAEs (62% vs. 41%) and more often discontinued treatment (62% vs. 41%) than patients without previous psychiatric disorders [84]. Similarly, Fernández-Bargiela et al. [75] tried to prove that a higher risk of discontinuation of DTG treatment is present in patients with mental disorders. Similar observations were made by Cusato et al. [85].

## Risk factors for the occurrence of neuropsychiatric disorders

In 2022, Cusato et al. revealed that patients receiving DTG, which inhibits the renally and neuronally expressed organic anion transporter 2 (encoded by *SLC22A2*), had neuropsychiatric symptoms. The effect of the SLC22A2 808C > A genetic variant in patients receiving DTG was evaluated and analyzed by real-time PCR. Among the 627 participants in the study, CA/AA carriers had a higher frequency of comorbid psychiatric illness and antidepressant use. Following 27.9 months of therapy, 108 participants discontinued DTG, with 64 having done so due to neuropsychiatric symptoms. Patients with a history of psychiatric comorbidities were more likely to discontinue DTG, whereas patients with the SLC22A2 CA/AA genotype were not. Within 30 days, most participants were completely symptom-free (61.8%). Discontinuation of DTG due to NPSAEs was not uncommon, and it was more common in participants with pre-existing psychiatric disorders. An interaction was observed between the SLC22A2 genetic variant and psychiatric comorbidities. Complete recovery from neuropsychiatric symptoms was not observed in 38.2% of patients after discontinuation of DTG, suggesting the involvement of additional factors [85].

Some researchers have looked for risk factors for the occurrence of central adverse events. DTG is metabolized by uridine diphosphate (UDP)-glucuronosyl transferase 1A1 (UGT1A1), and to a lesser extent by cytochrome P450 3A (CYP3A). Yagura et al. studied the relationship between a UGT1A1 gene polymorphism and the risk of neuropsychiatric adverse effects (*i.e.*, dizziness and headache, insomnia, restlessness, and anxiety) in 107 HIV-infected Japanese patients receiving DTG. Patients with one or two UGT1A1*6 and UGT1A1*28 alleles had a higher incidence of adverse events than those with normal alleles. Patients with abnormal alleles who were over 40 years of age had higher serum concentrations of DTG [86]. Other researchers have suggested that the increased risk of adverse effects with DTG use may be related to impaired mitochondrial function and cellular metabolic disorders [87].

As mentioned earlier, risk factors for neuropsychiatric disorders may also include older age, female gender, a history of neuropsychiatric disorders, and a negative human leukocyte antigen (HLA)-B*5701 test result. These factors have not been confirmed by all authors, and their relationship with the occurrence of treatment complications requires further observation [88].

A recent meta-analysis showed that there may be an increased risk of depression when DTG is co-administered with rilpivirine (RLP) compared to a single administration of either drug. The neurotoxic effect of DTG is explained by the ability of the drug to penetrate the BBB by passive diffusion, the possibility of changing the tight connections in the BBB, and secondary neuritis [89]. The authors refer to animal studies showing that DTG accumulating in the CSF can lead to oxidative stress and changes in neuronal hemostasis [42, 90]. According to other authors, the neurotoxic effect of DTG may also be associated with an increased concentration of DTG in the CSF, because, as already mentioned, the concentration of DTG positively correlates with the degree of neurotoxicity [44]. A potential mechanism for the increased toxicity of DTG when co-administered with RLP may be explained by drug–drug interactions with breast cancer resistance protein (BCRP), which is the main efflux transporter protein that removes drugs from inside cells. DTG is a BCRP substrate inhibited by RLP. Thus, increased DTG levels may potentially result from BCRP inhibition by RLP [91–94].

Neurotoxicity may also occur when DTG is co-administered with sertraline. Ma et al. showed that the interaction of these two drugs increases the permeability of the BBB and risk of NPSAEs [95].

## Changes in the summary of product characteristics

The Summary of Product Characteristics (SmPC) for Tivicay^®^ (ViiV Healthcare BV) has been updated several times as new information has become available. Initial results from animal studies show that single oral doses of DTG up to 500 mg/kg body weight in rats and 1000 mg/kg body weight in monkeys have minor effects on the nervous, respiratory, and cardiovascular systems. Already in the first characterization of the Tivicay^®^ medicinal product (13 November 2013) based on the SPRING-2, SAILING, and SINLGE studies, NPSAEs included headache, insomnia, fatigue, depression, and abnormal dreams [5]. The 12 November 2020 SmPC update was based on single case reports of NPSAEs, such as abnormal behavior, affective disorders, depression, insomnia, suicidal ideation, suicide attempts, and overdose, that occurred during study P1093 [96]. The current version of the SmPC is dated 18 November 2022. According to its provisions, central adverse reactions that are very common (≥ 1/10) and common (≥ 1/100 to < 1/10) include headache and insomnia, unusual dreams, depression, anxiety, and dizziness; uncommon (≥ 1/1000 to < 1/100) include panic attacks, suicidal thoughts, and suicide attempts (especially in those with a history of depression or mental illness), and rare (≥ 1/10,000 to < 1/1000) include suicide (especially in people with a history of depression or mental illness). Taking into account the number of HIV patients treated in Poland (16,000) and the fact that therapeutic regimens containing DTG are among the more widely recommended, there is a statistically low chance to observe NPSAEs, though they are reported by patients. It is important that doctors pay attention to the symptoms reported by patients and take a detailed history of insomnia or depressed mood and, discreetly, if possible, adjust the therapy to the patient’s needs [97].

## Clinical significance of observed CNS adverse effects

The publications cited above indicate that DTG is associated with CNS disorders that may lead to a discontinuation or change of treatment. In most cases, the factors predisposing to their occurrence and affecting their severity are unknown. Prospective studies that will enable the identification of a larger group of patients with neuropsychiatric disorders are justified and could identify factors predisposing to their occurrence. There are no data in the literature on the therapeutic interventions used, assessment of their effectiveness, impact on the frequency and severity of neuropsychiatric disorders, and the risk of treatment discontinuation. Such studies are necessary to precisely determine the need for treatment of neuropsychiatric disorders and indicate the optimal treatment. At the same time, new information on the safety of a drug, in this case, DTG, is a natural element associated with the presence of a new drug on the market. Clinical trials, although conducted very meticulously according to restrictive rules, are not able to provide full and exhaustive knowledge about the drug, especially in the context of its safety. One should keep in mind the limitations related to the number of patients participating in clinical trials, limitations of their duration, and strictly defined groups of patients who qualify for the trials in accordance with the inclusion and exclusion criteria. All this means that rare, distant adverse effects not directly related to the mechanism of action can be identified only at the post-registration stage. Therefore, the role of pharmacovigilance is essential in this matter. Importantly, the central adverse events identified for DTG do not preclude its use in clinical practice. The drug belongs to a basic group of antiretroviral compounds currently used in the treatment regimens of patients with HIV. The effectiveness of DTG has been proven in pre- and post-registration studies. The research is only to draw attention to the mechanism of selected adverse effects, explain their causes and possible risks, indicate the need to monitor patients for selected symptoms, and allow practitioners to use the drug safely and effectively.

## Conclusions

DTG is a first-line drug in HIV-positive patients used as part of combination therapy with other ATRs. It is recommended in two-drug regimens of similar efficacy and tolerability to three-drug regimens. The choice of DTG as a first-line drug is due to its rapid and effective reduction of viral titers in the blood. DTG has been shown to be a more effective drug, easier to take, and to have fewer adverse effects than current alternative drugs. DTG also has a high genetic barrier to the development of drug resistance, which is particularly important in patients who have developed resistance to other types of ARTs. The benefit of DTG therapy is the relatively good tolerability of the drug and the low risk of drug interactions, and it can be used in patients with tuberculosis which might be concomitant to HIV infection. DTG is used once daily, and such drug regimens improve patient compliance.

Undoubtedly, a limitation of treatment is the possibility of neuropsychiatric disorders, which may even lead to treatment withdrawal. The risk of adverse CNS effects is slightly higher for DTG than other INSTIs, but the intensity is generally mild to moderate.

## Data Availability

Data supporting Fig. 1 are publicly available as a part of a few preclinical studies evaluating the neurotoxicity of DTG: DOIs 10.3109/00498254.2014.942409 and 10.1093/jac/dkab501. Image data supporting Fig. 2, the Egan egg chart comparing the study drugs and selected commonly used drugs, are available as a part of the following publications: DOIs 10.1021/acs.jmedchem.5b00104, 10.1002/cmdc.201600182, 10.1093/jac/dkab501, and 10.3390/ph15050587. Data presented in Table 1 are available as a part of: DOIs 10.1093/ofid/ofz174, 10.1093/cid/ciu477, 10.1371/journal.pone.0006877, 10.1093/jac/dkt339, 10.1128/AAC.00507-10, 10.1089/aid.2015.0337, 10.1016/j.jpba.2020.113250, 10.1093/infdis/jiz624, 10.1093/jac/dkab334, 10.3947/ic.2021.0136, 10.1128/AAC.49.6.2504-2506.2005, 10.1124/dmd.108.020974, 10.1111/bcp.13552, 10.1097/QAD.0b013e3283489cb1, 10.1002/jcph.612, 10.1007/s13365-018-0626-4, 10.1128/AAC.06311-11, 10.1128/AAC.02329-12, 10.1093/jac/dkz504, 10.1097/QAD.0b013e328317a702, 10.1097/QAD.0b013e32814e6b1c, 10.1002/jcph.298, and 10.2174/1381612822666160726113001, as well as https://www.researchgate.net/publication/320879372_Cerebrospinal_Fluid_CSF_Concentrations_Efficacy_and_Neurocognitive_Effects_of_the_Combination_Of_Darunavir_Cobicistat_and_Rilpivirine_in_HIV-1_Naive_Adults. Data presented in Table 2 are available as a part of the following publications: DOIs 10.1111/hiv.12468, 10.1097/QAD.0000000000001590, 10.1136/ejhpharm-2020-002374, and 10.33448/rsd-v11i4.26250, and https://www.ema.europa.eu/en/documents/procedural-steps-after/tivicay-epar-procedural-steps-taken-scientific-information-after-authorisation_en.pdf, https://www.natap.org/2017/IAS/IAS_39.htm (Late Breaker Poster Abstract TUPDB0201LB).

## Abbreviations

CNS: Central nervous system
HIV: Human immunodeficiency virus
BBB: Blood–brain barrier
cART: Combined antiretroviral therapy
HAD: HIV-associated dementia
RNA: Ribonucleic acid
ATR: Antiretroviral drugs
DTG: Dolutegravir
INSTI: Second-generation integrase chain transfer inhibitor
FDA: Food and drug administration
DNA: Deoxyribonucleic acid
EMA: European medicines agency
RAL: Raltegravir
EVG: Elvitegravir
BIC: Bictegravir
CAB: Cabotegravir
TDF: Tenofovir disoproxil fumarate
FTC: Emtricitabine
IC_90_: 90% Inhibitory concentration
PSA: Polar surface area
BOILED-Egg: Brain or IntestinaL Estimate D permeation method
HIA: Human intestinal absorption
ABC: Abacavir
3TC: Lamivudine
ABCB1: ATP-binding cassette transporter protein P-glycoprotein
ABCG2: ATP-binding cassette transporter
PGP: P-glycoprotein PET: positron emission tomography
EC: Endothelial cell
CSF: Cerebrospinal fluid
NPSAE: Neuropsychiatric adverse events
ALT: Alanine aminotransferase
AST: Aspartate aminotransferase
CPK: Creatine phosphokinase
COBI: Cobicistat
TDF: Tenofovir disoproxil fumarate
ATV: Atazanavir
DRV: Darunavir
PI: Protease inhibitor
EFV: Efavirenz
NNRTI: Non-nucleoside reverse transcriptase inhibitor
LPV/r: Lopinavir/ritonavir
ATV/r: Atazanavir/ritonavir
DRV/r: Darunavir/ritonavir
DRV/c: Darunavir/cobicistat
PCR: Polymerase chain reaction
UDP: Uridine diphosphate
UGT1A1: Glucuronosyl transferase 1A1
CYP3A: Cytochrome P450 3A
(HLA)-B*5701: Human leukocyte antigen B*5701
RLP: Rilpivirine
BCRP: Breast cancer resistance protein
SmPC: Summary of Product Characteristics
